# Beneficial Effects of *Monascus* sp. KCCM 10093 Pigments and Derivatives: A Mini Review

**DOI:** 10.3390/molecules23010098

**Published:** 2018-01-03

**Authors:** Daehwan Kim, Seockmo Ku

**Affiliations:** 1 Laboratory of Renewable Resources Engineering, Purdue University, West Lafayette, IN 47907-2022, USA; kim1535@purdue.edu; 2 Department of Agricultural and Biological Engineering, Purdue University, West Lafayette, IN 47907-2093, USA; 3 Fermentation Science Program, School of Agribusiness and Agriscience, College of Basic and Applied Sciences, Middle Tennessee State University, Murfreesboro, TN 37132, USA

**Keywords:** bioconversion, derivatives, *Monascus* fermentation, microbial pigments, secondary metabolites

## Abstract

The production of *Monascus* pigments and related byproducts, via microbial fermentation, has been broadly utilized as coloring by traditional food industries and as a natural textile dye. In addition to these traditional purposes, *Monascus* pigments have been recently favored for a variety of commercial and academic purposes. Pigments and derivatives formed during *Monascus* fermentation have pharmaceutical and clinical properties that can counteract common diseases, including obesity, type-2 diabetes, and cancer. Various research attempts have investigated the optimum conditions for this derived compound synthesis, as well as the still-unknown bio-functional effects. Recently, several studies were conducted using *Monascus* sp. KCCM 10093 and its derivatives. These experimental outcomes potentially reflect the bio-functional features of *Monascus* sp. KCCM 10093. However, no publication to date provides an overview of *Monascus* sp. KCCM 10093’s unique metabolite products, functionalities, or biological pathways. In order to develop profitable commercial applications of *Monascus* sp. KCCM 10093, it is necessary not only to conduct continuous research, but also to systematically organize previous *Monascus* studies. The goals of this review are to investigate the current derivatives of *Monascus* sp. KCCM 10093 pigments—some of which have demonstrated newly-identified functionality—and the relevant uses of these molecules for pharmaceutical or nutraceutical purposes.

## 1. Introduction

In the past, food was used to eliminate hunger and meet nutritional requirements. In contemporary society, food is being used as a means to eliminate disease and improve our quality of life. Food consumers’ concerns regarding how foods and food supplements influence health have increased in recent years [1]. Functional food (also known as superfood, nutraceutical, and dietary supplements) comprises an actively growing business sector [2]. Therefore, the food markets of major developed countries are being driven by the concept of food functionality [3,4]. Food supplements are being used to provide additional essential nutrients, bio-functionality, and counteract health issues. The health-benefit characteristics of food supplements are expected to have a positive impact on worldwide industrial demand. The world functional food market was valued at $129.39 billion USD in 2015 [5]. Grand View Research estimates that the functional food market will steadily increase over the next eight years, due to growing consumer interest in healthy eating [6].

Among the various microbial-derived food subsidiaries, many East Asian providers have lately become interested in the application of edible fungi. In fact, this research trend is unsurprising [7]. Although the *Monascus* genus (filamentous fungus) was named in 1884 by van Tieghem, its various fermented foods and commodities have been used for thousands of years in East Asian countries and regions, including China, Korea, Indonesia, Okinawa, the Philippines, and Taiwan [8,9]. Traditionally, the people of East Asia have used a variety of fungi (e.g., *Actinomucor* spp., *Amylomyces* spp., *Rhizopus* spp., *Monascus* spp., *Neurospora* spp., *Aspergillus* spp., *Penicillium* spp., *Torulopsis* spp., *Trichosporon* spp., and *Zygosaccharomyces* spp.) [10] to produce various fermented food products. Food consumers have traditionally used fungi for food processing, which has led to the welcome addition of fungi into food. The *Monascus* fungus has been widely utilized for nearly two thousand years, as a natural food coloring agent, food antiseptic, and alternative medicine that eases digestion and soothes pain. *Monascus*-fermented red mold rice first appeared in a 1590 Chinese medicine book that detailed its use as a valuable coloring, flavoring, preservative, and therapeutic agent [11,12]. Red mold rice is synonymous with Hongqu, Honchi, Angkhak, and Red Chinese rice in China, Koji and Red Koji in Japan, Red mold in the U.S., and Rotschimmelreis in Europe [13,14].

**Figure 1:**
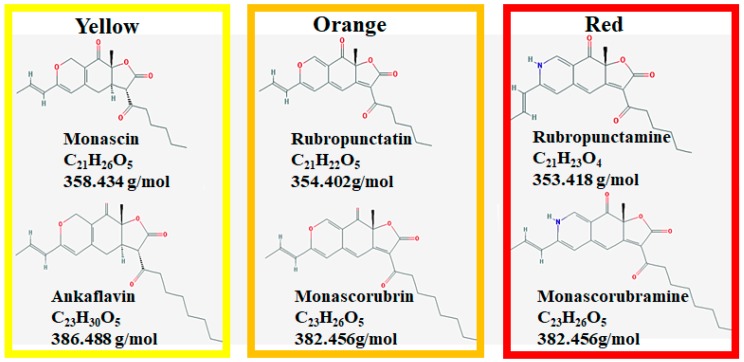
Six major *Monascus* pigments’ chemical structures.

It is reported that *Monascus* pigments can exist and be produced in a cell-bound form [15,16,17]. These products contain beneficial secondary metabolites, also called azaphilones [18]. *Monascus* can generate more than 90 distinct molecules [19]. There are six known *Monascus* pigments in three colors: orange (monascorubrin and rubropunctatin), yellow (ankaflavin and monascin), and red (monascorubramine and rubropunctamine) (Figure 1) [20].

*Monascus* growth and the production of its derived byproducts are easily subject to change based on culture conditions. For instance, octanoic acid, a medium-chain fatty acid, is essential to *Monascus* pigment color changes, via its chromophore site binding [21]. Previous works found that the trans-etherification of octanoic acid causes the formation of orange pigments; a decrease in orange pigments (mainly monascorubrin) generates yellow pigments, and the proliferation of orange pigments increases the production of red pigments [18,21,22,23]. Other studies reported that morphological change is a major contributor to cell growth and pigment production. Shin et al. [24] found that co-cultivations of *Saccharomyces cerevisiae* or *Aspergillus oryzae* in *Monascus* cultures increased cell growth and pigment yield, likely due to the hydrolytic enzymes produced from *S. cerevisiae* or *Asp. oryzae*. Hydrolytic enzymes, such as amylase and chitinase, caused cell wall degradation and morphological change in *Monascus,* which may have induced the cells to produce protective molecules (e.g., hydrophobic pigments) to defend against cellulolytic enzyme attacks. Further work done by Ju et al. [25] optimized red pigment production, by controlling chitinase activity during *Monascus* fermentation. The addition of an *S. cerevisiae* culture-filtered solution (containing chitinase) and maintained chitinase activity improved cell mass and pigment concentration. Kim et al. [26] also demonstrated that morphological control of *Monascus* in seed cultures augmented the pigment production in scale-up performances, from a 5 L to 300 L reactor, by controlling impeller tip speed and oxygen transfer rate (K_L_a). Moreover, *Monascus* pigment concentrations, color profiles, and chemical properties are highly related to the particular strain [27], medium substrate [28,29,30], nitrogen and oxygen concentrations [29,31,32], pH [33,34], temperature [35], and mixing conditions [35,36]. Over 30 *Monascus* strains have been documented. Three strains (*M. Pilosus, M. purpureus,* and *M. ruber*) have been studied extensively and applied in industry [15]. The conventional *Monascus* species used as food coloring agents in Asian countries as well as universally-perceived genera are summarized in Table 1.

Over the past few decades, considerable attention has been paid to *Monascus*-fermented products and secondary metabolites, due to their therapeutic effects on a variety of ailments. These secondary compounds include edible pigments, isoflavones, enzymes, fatty acids, organic acids, dimerumic acid (antioxidant), vitamins, γ-aminobutyric acid (GABA, hypotensive agent), monacolin K (lovastatin, anti-hypercholesterolemic agent), and other active components.

According to the Food and Drug Administration (FDA), “a new dietary ingredient is a dietary ingredient that was not marketed in the United States in a dietary supplement before 15 October 1994” [54]. However, no list of the dietary supplements on the market, or on the market as dietary supplements before 15 October 1994, exists. There is no way for the industry to verify this timestamp. Despite the fact that a variety of bio-functional secondary metabolites are produced by *Monascus*, the only *Monascus* sp. product that is commercially marketed and approved by the FDA’s Federal Food, Drug, and Cosmetic Act is *Monascus* 8000F [54]. The FDA announced the “New Dietary Ingredients Used in Dietary Supplements” in August 2016, which states, “Probiotics and other microbial ingredients do not constitute a distinct category of dietary ingredient” [6]. The document also states that algae and fungi do not fall into the category of microbial ingredients. Although this is not yet legally binding information, many hurdles to the production and application of fungi can be expected as a result of this FDA statute. The corresponding research investigations into exactly how certain strains accurately produce secondary metabolites, and how the produced secondary metabolites are biologically converted, are prioritized projects in the edible molds field, as well as other microbial studies (e.g., *Lactobacillus* sp. and *Bifidobacterium* sp.) [55]. Because the functional materials produced by the *Monascus* strain are distinct, and the pigment activity depends on the growth mode, numerous research fields would benefit from the *Monascus* applications currently in development [56]. For instance, various biocatalyzed *Monascus* pigments obtained from *Monascus* sp. KCCM 10093 fermentation were combined with supplements, and tested to observe their functional effects. The functional effects of KCCM 10093 derivatives produced from fermented pigments include obesity inhibitory activity [57], cholesteryl ester transfer protein (CETP) inhibitory activity [58], hepatitis C virus replication inhibition [59], diet-related lipase and α-glucosidase inhibitory activities [60], anti-atherosclerosis effects [20], antibacterial activity [61,62], and photo-stability [22,23,37]. Although several studies have reported the amino acid or amine derivatives of *Monascus* sp. KCCM 10093 pigments and their benefits, to the best of our knowledge there is no existing review of these studies. The main purpose of this review is to summarize our current understanding of KCCM 10093 fermented byproducts (mainly derivative compounds), their valuable natural effects, and their potential industrial applications.

## 2. Antimicrobial and Antiviral Effects of *Monascus* Pigment Derivatives

**Figure 2:**
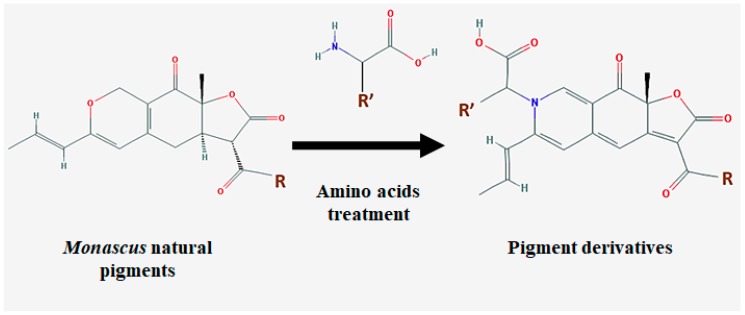
Substitution of *Monascus* pigments’ oxygen moiety for amino acids’ nitrogen moiety. R = C_5_H_11_ or C_7_H_15_. R’ is a functional group of amino acids.

*Monascus* pigment products are produced via solid state fermentation (SSF), and the inoculation of the seed culture (the dry form of *Monascus*) onto specific solid substrates, such as agar medium, bread, rice bran, cassava, apple, pomace, and wheat [36,63,64,65]. Enhanced pigment and other secondary metabolite production is achieved with the supplementation of other compounds, such as amino acids, amines, amino sugars, amino alcohols, nitrogen compounds, nucleic acids, chitosan, and co-culture with either a *Saccharomyces cerevisiae* or *Aspergillus oryzae* strain [24,66]. Interestingly, the co-culture with one of these strains in the solid medium caused remarkable morphological changes in *Monascus* cells. This is possibly due to the production of hydrolytic enzymes (such as α-amylase and chininase) from either from *S. cerevisiae* or *A. oryzae*. These enzymes could damage the cell walls of *Monascus*, and induce the cells to produce more hydrophobic molecules (e.g., pigments) as a mechanism to protect themselves from hydrolytic enzymes [24]. When *Monascus* is cultured with supplementary molecules, the pigments’ oxygen group can be substituted by supplements, which may benefit the generation of synthesized, value-added biomolecules (Figure 2) [20].

The cultivation protocol for derivative synthesis is as follows: (1) slant agar culture, (2) seed culture, (3) inoculation of seed culture, (4) main cultivation with supplements, (5) isolation, and (6) drying. Youngsmith [16,17,67] reported that the synthesis of *Monascus* pigments and metabolites can be determined by the *Monascus* growth. Since fungi consume energy sources (carbon and nitrogen) from growth substrates during the initial stage, the synthesis of secondary compounds depends upon their growth conditions. This indicates that the substrate composition and the management of fungal growth are major indicators of desirable byproduct production.

The recent use of non-chemical preservatives, minimally-processed materials, and natural food ingredients are crucial consumer demands, to avoid the toxicological issues related with artificial food antimicrobials [68,69,70]. *Monascus* pigments and their derivatives have been utilized in food coloring and traditional additives; some have even been studied and applied in food industries. The antimicrobial activity of fermented pigments has a promising potential for food alternatives, since these compounds are practically natural and free from chemical synthesis. *Monascus* pigments fermented by *Monascus ruber* CCT 3802 [28,32,36], *Monascus purpureus* [71], *Monascus purpureus* N11S [72], and *Monascus* M3428 [73] were examined to investigate if they demonstrate antimicrobial activity in response to *Escherichia coli*, *Salmonella enteritidis*, *Bacillus subtilis*, *staphylococcus aureus*, and yeast, respectively. Additional studies on the antimicrobial effects of *Monascus* fermentation reported that gram-positive bacteria are more susceptible to inhibition than gram-negative bacteria, and *Latobacillus*‘ resistance is negligible [74,75,76]. This result encouraged the use of a *Monascus* pigment as a replacement for nitrite in meat production [77]. The specific mechanisms and properties of this finding are still unknown; however, the finding suggests that antimicrobial activity is significantly determined by cell growth conditions and culture medium compositions [72,73,78].

Kim et al. [61] found that the antimicrobial activity of the red pigments produced from KCCM 10093 fermentation produced poor responses to gram-positive bacteria, gram-negative bacteria, and filamentous fungi. However, these pigment derivatives (which were cultivated with amino acids) displayed dramatically improved antimicrobial activities during minimum inhibitory concentration (MIC) tests. The amino acid derivatives containing a phenyl group demonstrated strong antimicrobial activities. Further testing of Phe and Tyr derivatives (with a phenyl ring) presented higher activity (MIC: 4–16 μg/mL) than the orange pigment (MIC: 10–30 μg/mL) [71,79] and the red pigment (MIC > 32 μg/mL), which was mainly due to the hydrophobicity and absorption of the pigment derivatives. These pigment derivatives may affect cell morphology by aggregating the cells, which results in suppressed cell growth, due to inhibited oxygen transfer and nourishment [61]. Additional studies reported [80,81] that chitosan had antimicrobial activities, via binding or forming cell pellets, which resulted in a lack of oxygen transfer. It is worth noting that chitosan’s antimicrobial activities were only effective with gram-negative bacteria and yeast with high MIC values (MIC: 100–10,000 μg/mL).

Chronic liver disease caused by the hepatitis C virus (HCV) is a global health concern. More than 170 million HCV infections may result in HCV-related diseases, including chronic hepatitis and hepatocellular carcinoma [82,83]. Many studies have investigated HCV infection prevention, such as the tests with pegylated interferon (IFN) and ribavirin (a nucleoside analogue) developed for clinical trials. However, further research and other anti-HCV drugs are required, due to the side effects from the use of IFN-α and ribavirin, as well as low virological sensitivity (<50% for HCV genotype 1 or 4) [84]. Since the HCV NS5B RNA-dependent RNA polymerase (RdRp) enzyme is considered a key enzyme of HCV replication, the inhibition or deactivation of this enzyme is an effective strategy against viral replication. Sun et al. [59] have identified that *Monascus* pigment derivatives from KCCM 10093 demonstrate antiviral activity that could remarkably suppress HCV RNA replication. In the Huh7 human hepatoma cell line, six major orange pigment derivative incubations at 10 μM inhibited HCV RNA levels by 38–53%. The positive control (standard HCV therapy) of IFN-α (100 IU/mL) decreased the HCV level by 75%. Further tests with the combination of screened *Monascus* pigment derivatives (Phe, Val, and Leu) and IFN-α increased the inhibition levels by up to 88%, and the Leu derivative demonstrated the most effective anti-HCV activity. HCV RNA inhibition via this combination treatment was dose-dependent, with an IC_50_ value of 8.9 μM, which did not affect cell viability at dose concentrations up to 50 μM (no cytotoxicity). The HCV inhibition mechanism is not yet clearly identified; however, further studies on HCV RNA replication suggested that *Monascus* pigment derivatives blocked the cholesterol biosynthetic steps of mevalonate, as well as 3-hydroxy-3-methyglutaryl coenzyme A (HMG-CoA) reductase, which are important intermediate compounds that synthesize cholesterol as an end product. In addition, the antiviral activity of *Monascus* pigment derivatives was verified with other types of HCV (genotypes 2a and 1b), which could offer potential alternatives to current HCV therapies and antiviral drugs.

## 3. Regulation of Cholesterol Synthesis and Anti-Obesity Effects

Atherosclerosis—the buildup of oxidized cholesterol, fat, calcium, waste products, and other molecular substances in the arteries—is a significant disease worldwide. It is reported that atherosclerosis accounts for around 32% of deaths in the U.S. [85,86]. As undesirable molecules build up, also called plaque, the diameter of the artery declines, and the arterial walls thicken, which constricts the supply of oxygen and oxygen-rich blood to organs and cells throughout the body. Furthermore, plaque can accumulate in other arteries throughout the body (i.e., the heart, muscle, brain, leg, and kidney). Blood clots caused by plaque may travel to another part of the body and inhibit blood flow as well. These plaque deposits cause different atherosclerosis-related diseases, including coronary heart disease, coronary microvascular disease, stroke, carotid artery disease (plaque in the neck arteries), peripheral artery disease (plaque in the arteries of the legs, arms, and pelvis), and chronic kidney disease [87,88]. Decreasing low-density lipoprotein cholesterol (LDL-C) or increasing high-density lipoprotein cholesterol (HDL-C) can prevent atherosclerotic diseases [89,90,91]. Cholesteryl ester transfer protein (CETP) is the main molecule that transfers cholesteryl esters (CEs) and triglycerides (TGs) from HDL to TG lipoproteins, which contributes to not only increased LDL levels, but also reduced HDL levels [92,93]. The inhibition of CETP is one of the most effective approaches to manage the cholesterol levels of the body. Jang et al. [58] reported that amino acid derivatives of KCCM 10093-fermented pigments had an inhibitory effect on CETP. Their study showed that 19 different amino derivatives were generated from the fermentations of orange pigments (monascorubrin and rubropunctatin) and l-amino acids. Initial CETP inhibition testing of the 19 derivatives at 0.5 μM treatment showed that 14 of the derivatives had inhibitory activities. Among them, l-Thr and l-Tyr derivatives displayed the highest CETP inhibitions of 20% and 13%, respectively. The amino acid is strongly related to CETP inhibition. The amino acid phenyl structure is essential to inhibition. During further tests of these compounds, ranging from 0.2–2 μM, CETP inhibitions were observed of up to 55% with l-Thr, and 44% with l-Tyr derivatives at 2 μM, respectively, suggesting that a high dose of these derivatives increases the CETP inhibition level. The IC_50_ values of l-Thr and l-Tyr derivatives were estimated to be about 1.0 μM and 2.3 μM, respectively.

Some drugs are prescribed to reduce atherosclerosis, such as torcetrapib (against hypercholesterolemia), anacetrapib (to treat hypercholesterolemia), and evacetrapib (to inhibit CETP). These chemical drugs have IC_50_ values of 5–50 nM, and lead to decreased LDL-C (15–40%) and increased HDL-C (55–140%) [94,95,96]. Other findings described that natural molecules from fruits have CETP-inhibitory activities; for instance, polyphenols from apples and grapes (IC_50_ at 100 mM) and xanthohumol (IC_50_ at 88 μM) [97,98,99]. While the IC_50_ values of the l-Thr and l-Tyr derivatives were lower than the drug values, these natural compounds could be proposed as alternatives to synthetic medications. Furthermore, the chemical drugs used in clinical treatment have toxicity problems. *Monascus*-fermented derivatives are natural molecules, and considerably less toxic [96,100].

Cholesterol biosynthesis is the result of transferring acetyl-CoA to cytosol. 3-hydroxy-3-methyglutaryl coenzyme A (HMG-CoA) reductase is a key enzyme, able to convert HMG-CoA to mevalonate, and consequently increase cholesterol levels [101,102]. Several studies have addressed that the inhibition of HMG-CoA reductase could reduce cholesterol levels in the human body. Moreover, a number of commercial drug clinical trials (i.e., Lovastatin, Monacolin K, and other HMG-CoA inhibitors) regulate the elevated cholesterol levels caused by hypercholesterolemia [103,104,105]. *Monascus* pigment derivatives produced from KCCM 10093 have been reported to hamper HMG-CoA reductase, and further anti-cholesterol tests were performed in vivo using mice [20]. Among the 13 amino acid derivatives that were developed, the Thr-derivative had the highest HMG-CoA inhibition at 35%, similar to the orange pigment (36%). The oxygen moiety of the pigment is susceptible to substitution from the nitrogen moiety in the supplemented amino acid during fermentation. The oxygen moiety of the orange pigment and its derivatives can increase HMG-CoA reductase inhibition, by biding onto the enzymes. Similar to the findings of previous works [57,60], the phenyl groups (aromatic, hydrophobic, and non-polar aliphatic) of *Monascus* pigment derivatives correlated to enzyme inhibitions. Further diet effect tests in vivo presented that the control group (diet medium +2% (*w*/*w*) cholesterol) increased mice weight by 27–51%, while the supplementation of 0.01% orange pigment (g pigment/g mouse weight) or 0.02% Thr-derivative (g/g mouse weight) in similar experimental conditions did not affect weight changes in mice. In addition, feeding cholesterol to the mice increased HDL and LDL by 41% (from 38.6 to 54.5 mg/dL) and 110% (from 47.2 to 99.2 mg/dL), respectively. The Thr-derivative increased HDL by 1–9%, and decreased LDL by 28–26%. These results suggested that the orange pigment and the Thr-derivative have anti-atherosclerosis effects, and could be considered as natural alternatives used in clinical trials.

Overweight and obese conditions often lead to various chronic diseases, and can increase the risk of obesity-related diseases such as high blood pressure (hypertension), dyslipidemia, coronary heart disease, stroke, gallbladder disease, osteoarthritis, mental disorders (clinical depression, anxiety, and depression), cancers (colon, liver, kidney, breast, and endometrial), and sleep and breathing problems [106]. The medical care cost of obesity and obesity-related diseases was estimated to be about $147 billion USD in 2008. Worldwide, $3.38 billion USD ($79 per obese individual) and $6.38 billion USD ($132 per obese individual) has been spent on obesity and obesity-related diseases, respectively, and these costs are increasing [106,107].

**Figure 3:**
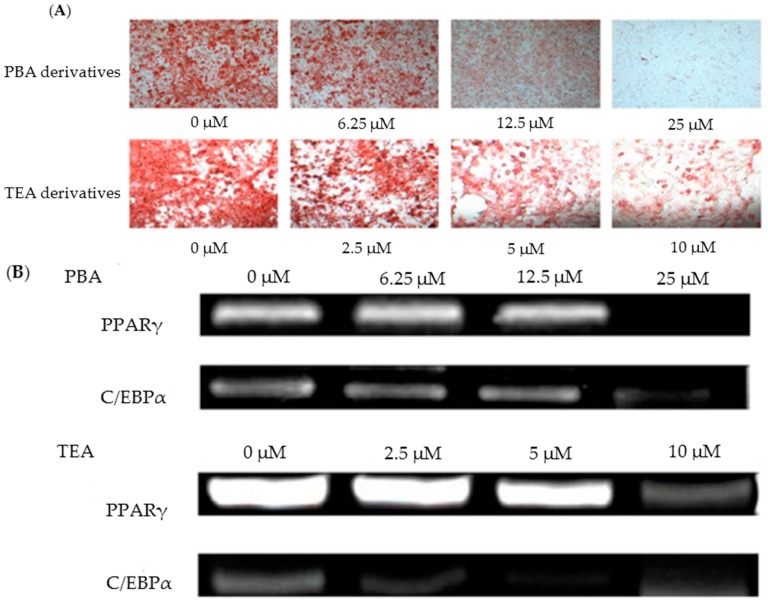
(**A**) Lipid cells treated with varied levels of amine derivatives (dyed with oil red O); (**B**) Inhibited adipogenic transcriptional factors (PPARγ and C/EBPα), with various amine derivative concentrations (adopted and modified from Choe et al. [57]).

Choe et al. [57] found that amine derivatives of *Monascus* pigments demonstrated anti-obesity activities both in vivo (3T3-L1 preadipocyte mouse cells) and in vitro (C57BL/6 mice). *Monascus* pigments were initially produced via KCCM 10093 fermentation, and the isolated orange pigments were serially synthesized with 47 different amines. Among the 47 different amine derivatives, 16 derivative compounds were confirmed to have an inhibitory effect on adipogenic differentiation in 3T3-L1 cells. In particular, 4-phenylburylamine (PBA) and 2-(*p*-toyly) ethylamine (TEA) showed the best results, with about 40% reduction of cell differentiations at 2.5 μM and 12.5 μM concentrations, respectively, compared to the control test without derivatives (Figure 3A) [57]. To better understand these inhibitory effects, further testing of PBA and TEA derivatives was conducted on the adipogenic transcriptional factors (PPARγ and C/EBPα). As the concentrations of the PBA and TEA derivatives increased, the expression levels of PPARγ and C/EBPα decreased. For PPARγ, 70% and 80% inhibition was observed with treatments of PBA (12.5 μM) and TEA (10 μM) derivatives, respectively (Figure 3B) [57].

C/EBPα tests, with similar PBA and TEA derivative concentrations and experimental conditions, resulted in 70% transcriptional inhibition. In additional in vivo tests, mice were fed PBA and TEA derivatives at different feeding concentrations for 16 weeks, to examine the effects of derivatives on weight gain, epididymal adipose tissue (EAT), and liver tissue. The supplementation of derivatives at 0.1–0.4 mg/g mouse per day to the high-fat diet (HFD) group remarkably decreased the weight gain by 26.8–59.5%, compared with the results from the HFD group without the derivative supplements. The increase of derivative concentrations caused a consistent pattern of inhibitory effects and decreased weight gain. TEA derivatives of 0.4 mg/g mouse per day showed the lowest weight gain of 59.5%. Similarly, decreased EAT and liver tissue weights were observed with the derivatives; TEA derivatives of 0.2 and 0.4 mg/g mouse per day reduced EAT weight and liver tissue weight by 56% and 9.8%, respectively. These results indicate that amine derivatives of *Monascus* pigments have valuable anti-obesity effects. The exact mechanisms and activities of derivatives should be examined through future research. These previous works may suggest that the phenyl group of amine derivatives, a cyclic atom ring functional group, plays a key role in hindering the differentiation of plaque cells. Some studies have concurred with these results, and offered evidence that phenolic compounds—flavonoids, genistein, naringenin, rutin, EGCG, and curcumin—could contribute toward the inhibition of 3T3-L1 cell differentiation and adipogenic transcriptional factors [108,109,110,111].

## 4. Conclusions and Research Outlook

Based on the previous literature reviewed herein, *Monascus* pigments and their derivatives demonstrate promising potential in various applications: (1) color agents and food additives, (2) antimicrobials, (3) anti-virologicals, (4) anti-obesity, and (5) cholesterol regulation. The food preservative effect of *Monascus* pigments has been scientifically confirmed. Moreover, a commercial dietary product (Monacolin by Maruzen Pharmaceuticals) containing *Monascus* extract has been introduced in Japan [76]. As the interest in and applications of *Monascus* metabolites have grown, concerns regarding their potential toxicity and safe consumption have also increased. Citrinin, the dominant mycotoxin produced by *Monascus*, is able to contaminate *Monascus*’ secondary metabolites. Sabater-Vilar et al. [14] identified the citrinin in commercial *Monascus* samples, with concentration levels of 0.2–1.71 ug/g. Citrinin may cause organ damage in the liver, kidney, and organ tissues, and has been evaluated as a carcinogenic compound [13,112]. The production and toxicity of citrinin are highly relative to strain, media, carbon and nitrogen concentration, and temperature [21,72,113,114]. In response to the toxicity and safety issues, the maximum citrinin allowed in food supplements (based on red yeast *Monascus*) in Japan is 200 ng/g, and in the European Union the maximum is 2 mg/kg [115,116]. To the best of our knowledge, there is no research on safety, fungal toxin, and negative health effects of *Monascus* sp. KCCM 10093. Therefore, future studies should explore ways to experiment with lower levels of these possibly toxic compounds, and produce high-quality secondary metabolites via optimal fermentation processes. Other research groups have paid attention the biological activities of *Monascus* pigments for applications in iatrical treatments and cancer prevention. *Monascus*-fermented products have proven effective in a number of such therapies. Lee et al. [117] confirmed that ethanol extract from red mold rice (RMRE) was beneficial to Alzheimer’s disease prevention. Some works indicated that red mold rice and dioscorea exhibit anti-cancer activities in the skin [118], liver [119], colon [120,121], lungs [122,123], and mouth [124,125,126]. Along with these clinical evaluations of *Monascus* pigments and metabolites, further studies of its molecular biology began in the early 2000s. Molecular techniques for the transformation of *Monascus* include electroporation [127,128], polyethylene glycol-mediated transformation (PEG-MPT) [129], and restricted-enzyme mediated integration (REMI) [130]. In order to understand the key secondary metabolic steps in *Monascus* fermentation, specific genes for pigments, citrinin, G-protein, and monacolin K were examined and analyzed [131,132,133,134,135,136]. Although these genetic tools could facilitate our understanding of *Monascus*, the metabolite synthesis and biochemical pathway processes remain unclear. There are unknown novel ingredients and salutary effects of *Monascus* metabolites. Further investigation of *Monascus*, providing taxonomic comparison, phylogenetic analysis, and morphological and physiological properties, will improve its efficacy in therapeutic and food-based applications.

## Figures and Tables

**Table 1:** Summary of *Monascus* species utilized to produce fermented pigments.

Species	Strain	Medium	Color	Reference
*Monascus* sp.	KCCM 10093	Chemically defined medium	Red, orange	[22,23,37]
*Monascus* sp.	KB20M10.2	GPMY (chemically defined medium)	Yellow	[17,38]
*Monascus* sp.	ATCC 16436	MP I, II and III (chemically defined medium)	Orange, yellow	[8,39]
*Monascus* sp.	J101	Chemically defined medium	Red, yellow	[24,25,40]
*Monascus* sp.	B683	Chemically defined medium	Red, yellow	[41]
*Monascus* sp.	TTWMB 6093	Chemically defined medium	Red	[42]
*Monascus kaoliang*	ATCC 26264	Solid culture medium	Orange, yellow	[9]
*Monascus bisporus*	ATCC 36964	319 ^1^	Yellow	[43]
*Monascus eremophilus*	ATCC 62925	319 ^1^	Orange	[44]
*Monascus floridanus*	ATCC 64205	336 ^1^	Orange	[44]
*Monascus lunisporas*	ATCC 204397	319 ^1^	Orange	[45]
*Monascus sanguineus*	ATCC 200613	325 ^1^	Red	[46]
*Monascus pilosus*	ATCC 16363	325 ^1^	Orange	[44]
*Monascus ruber*	ATCC 15670	336 ^1^	Orange	[31]
*Monascus ruber*	ATCC 96218	Chemically-defined medium	Red	[21]
*Monascus ruber*	CCT 3802	Chemically-defined medium	Red, orange, yellow	[28,32,36]
*Monascus ruber*	102w	Chemically-defined medium	Red	[47]
*Monascus ruber*	LEB A 1-3	PDA ^2^	Red	[48]
*Monascus purpureus*	ATCC 16365	325 ^1^	Orange	[44]
*Monascus purpureus*	IMI 210765	PDA ^2^	Red, yellow	[49]
*Monascus purpureus*	NRRL 1992	PDA ^2^	Yellow	[50]
*Monascus purpureus*	CCM8152	Chemically-defined medium	Red	[51,52]

^1^ ATCC designed medium [53]; ^2^ Potato dextrose agar.

## Abbreviation

FDA	Food and Drug Administration
